# A Siamese tracker with “dynamic–static” dual-template fusion and dynamic template adaptive update

**DOI:** 10.3389/fnbot.2022.1094892

**Published:** 2023-01-11

**Authors:** Dongyue Sun, Xian Wang, Yingjie Man, Ningdao Deng, Zhaoxin Peng

**Affiliations:** School of Mechanical Engineering, Hunan University of Science and Technology, Xiangtan, China

**Keywords:** object tracking, Siamese network, template update, dynamic–static dual-template fusion, deep learning

## Abstract

In recent years, visual tracking algorithms based on Siamese networks have attracted attention for their desirable balance between speed and accuracy. The performance of such tracking methods relies heavily on target templates. Static templates cannot cope with the adverse effects of target appearance change. The dynamic template method, with a template update mechanism, can adapt to the change in target appearance well, but it also causes new problems, which may lead the template to be polluted by noise. Based on the DaSiamRPN and UpdateNet template update networks, a Siamese tracker with “dynamic–static” dual-template fusion and dynamic template adaptive update is proposed in this paper. The new method combines a static template and a dynamic template that is updated in real time for object tracking. An adaptive update strategy was adopted when updating the dynamic template, which can not only help adjust to the changes in the object appearance, but also suppress the adverse effects of noise interference and contamination of the template. The experimental results showed that the robustness and EAO of the proposed method were 23% and 9.0% higher than those of the basic algorithm on the VOT2016 dataset, respectively, and that the precision and success were increased by 0.8 and 0.4% on the OTB100 dataset, respectively. The most comprehensive real-time tracking performance was obtained for the above two large public datasets.

## 1. Introduction and motivation

Video object tracking, which refers to continuously tracking the state of an object in subsequent frame sequences by using the initial position and scale information of the object, is the basis for high-level visual tasks such as visual inspection, visual navigation, and visual servo (Nousi et al., 2020; Wang et al., 2020; Karakostas et al., 2021; Sun et al., 2021). In engineering practice, interference such as changes in the posture and scale of the object, noise interference, background occlusion, or variation of light conditions may lead to tracking failure, so object tracking remains a challenging task (Zhang et al., 2020; Zhang H. et al., 2021; Liu et al., 2022).

Object tracking methods can be roughly divided into generative methods and discriminative methods (Zhang Y. et al., 2021; Dunnhofer et al., 2022). Generative methods first build the model of the object and then search for the area which is most similar to the object in subsequent frames through iterating to achieve target positioning. Discriminative tracking algorithms transform the object tracking problem into a binary classification problem about the object and the background, and find the predicted object position by training a classifier to distinguish the object from the background. In general, generative methods do not rely on training samples and are easy to implement, while discriminative ones are stronger in robustness.

To handle various challenges, a significant amount of research has been focused on visual tracking in recent years. With the continuous advancement of machine learning and signal processing technology, algorithms based on correlation filters (Zhang J. et al., 2022; Ly et al., 2021) and deep learning (Haisheng et al., 2019; Voigtlaender et al., 2020; Tan et al., 2021; Zhang X. et al., 2022) have gradually replaced traditional methods as the mainstream object tracking algorithms. Both of these methods are discriminative methods. The basic idea of correlation filter tracking is to use a designed filter template for the correlation operation with the target candidate area, and the position where the maximum output response is located is the target position of the current frame. Before the advent of object tracking algorithms based on correlation filters, all tracking operations were completed in the time domain with a large amount of data and a long period of calculation time. However, the object tracking algorithms based on correlation filters convert the operation from the time domain to the frequency domain, reducing the amount of computation while ensuring the integrity of data. Early correlation filter object tracking algorithms include the MOSSE (Minimum Output Sum of Squared Error Filter) algorithm (Bolme et al., 2010), the CSK (Circulant Structure with Kernels) algorithm (Henriques et al., 2012), the KCF (Kernelized Correlation Filter) algorithm (Henriques et al., 2015), and the SAMF (Scale Adaptive Multiple Feature) algorithm (Li and Zhu, 2014), etc. All the above algorithms use manual features. Later, people began to introduce deep learning into correlation filtering, mining more robust depth features from the original data to replace traditional manual features, thereby further improving the robustness of correlation filtering algorithms. Typical object tracking algorithms which combine correlation filtering with deep learning include the Deep SRDCF (Convolutional Features for Correlation Filter Based Visual Tracking) algorithm (Danelljan et al., 2015a), the C-COT (Continuous Convolution Operators) algorithm (Danelljan et al., 2016a), and the ECO (Efficient Convolution Operators for Tracking) algorithm (Danelljan et al., 2017a), etc.

The deep learning object tracking algorithm based on the Siamese network has received extensive attention due to its good performance in testing various benchmark tracking datasets. SiamFC (Fully Convolutional Siamese Networks for Object Tracking; Bertinetto et al., 2016b), an earlier object tracking algorithm based on a fully convolutional Siamese network, uses a fully convolutional network structure to learn the similarity measurement between the target area and the search area, thus viewing tracking as a problem of searching for target objects across the entire image. Based on SiamFC, Bo et al. (2018) proposed SiamRPN (High Performance Visual Tracking with Siamese Region Proposal Network), an object tracking algorithm based on the region proposal network. The method, with a classification network for foreground and background estimation and a regression network for anchor bounding box correction included, estimates the position and size of the object through a bounding box with a variable aspect ratio, so that a more accurate bounding box can be obtained. Zhu et al. (2018) proposed a Siamese network tracking algorithm based on distractor aware, DaSiamRPN (Distractor-Aware Siamese Networks for Visual Object Tracking), which improves the discrimination ability of the model by introducing a distractor-aware module. Based on SiamFC or SiamRPN, Zhang and Peng (2019) proposed the SiamDW (Deeper and Wider Siamese Networks for Real-Time Visual Tracking) algorithm, which further improves tracking accuracy and robustness by introducing internal clipping units of residual blocks into deeper and wider networks. The SiamMask (Fast Online Object Tracking and Segmentation: A Unifying Approach) algorithm proposed by Wang et al. (2019) can simultaneously implement video object tracking and video object segmentation, and a predictive bounding box of an adaptive mask can be obtained during object tracking, which greatly improves the accuracy of tracking.

The above tracking algorithms based on the Siamese network achieved the optimal performance at that time, but all these algorithms use the position of the object in the first frame image as a fixed template throughout the tracking process, which makes them unable to deal well with the adverse effects of a change in the appearance of the object. In response to this problem, a template update mechanism was introduced into object tracking. Danelljan et al. (2015b) improved tracking efficiency by building a Gaussian hybrid model of the training sample together with a conservative template update strategy (updated every five frames). Galoogahi et al. (2017) proposed a background-aware correlation filter algorithm that achieves object tracking with high accuracy and real-time performance by intensively extracting real negative samples from the background to update the filter. The above studies use linear interpolation for template updating, which are prone to causing tracking drift, resulting in tracking failures. The UpdateNet (Learning the Model Update for Siamese Trackers) algorithm proposed by Zhang et al. (2019) implements template tracking through a trained convolutional neural network, which greatly improves the tracking performance. Wang W. et al. (2020) introduced a sparse update mechanism in the tracking framework, which could help adaptively select the appropriate level for object tracking. Such an update strategy reduces the complexity of the model to a certain extent. Huang et al. (2019) proposed a correlation filter tracker based on transfer learning, which updates the model by migrating historical filter data to improve the robustness of the tracker. Han et al. (2020) proposed a spatial regularization update method with content perception, which adjusts the weight distribution map by optimizing the constraint problem to better adapt to the changes in the object and background, so that reliable tracking can be achieved. Zhang Y. et al. (2022) proposed a dual-stream collaborative tracking algorithm combined with reliable memory updates, which realizes real-time tracking speed and a superior tracking performance.

The introduction of the template update mechanism effectively improves the performance of the Siamese network object tracking algorithm. However, there are various noise interferences in actual application situations. The template update mechanism brings new problems while better adapting to the changes in the appearance of the target, causing the template to be polluted by noise interference (Su et al., 2022).

In order to further improve the performance of object tracking algorithms, a Siamese tracker with the “dynamic–static” dual-template fusion and dynamic template adaptive update is proposed based on the DaSiamRPN and UpdateNet template update network in this paper. The new method combines a static template and a dynamic template that is updated in real time for object tracking. An adaptive update strategy is applied when updating the dynamic template, which helps decide whether to update the dynamic template by using the similarity between the tracking result of the current frame and the dynamic template, as well as the no reference evaluation results of the current frame image, to judge the necessity of updating the template and the possibility of template pollution if the template is updated. In this way, it can suppress the adverse effects of noise interference and pollution of the template while adapting to changes in the object appearance. The new method achieved the best comprehensive and real-time tracking performance on the two large public datasets, VOT2016 and OTB100.

## 2. Proposed method

**Figure 1:**
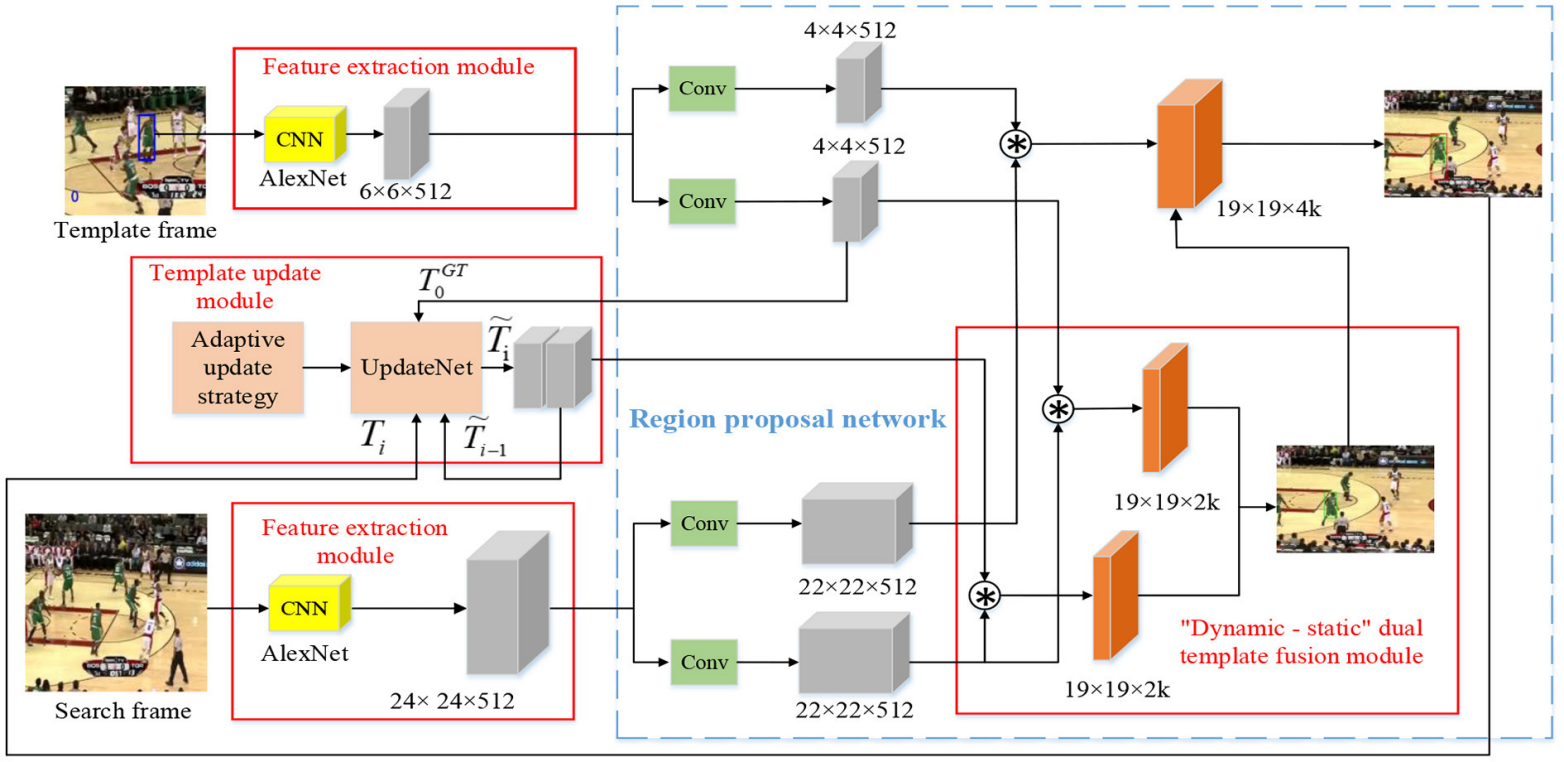
The overall network architecture.

Based on the DaSiamRPN algorithm, the proposed method introduces the “dynamic–static” dual-template fusion and dynamic template adaptive update mechanism. Its overall network architecture is shown in Figure 1, including the feature extraction module, RPN (Region Proposal Network), dynamic template update module, and the “dynamic–static” dual-template fusion module. In order to ensure the real-time performance of target tracking, the AlexNet shallow network used by the DaSiamRPN algorithm was also adopted by the feature extraction module. RPN consists of a classification network for foreground-background estimation and a regression network for anchor bounding box correction. It uses a bounding box with variable aspect ratio to estimate the position and size of the object, thus obtaining a more accurate bounding box. Based on the UpdateNet network, the dynamic template update module designed an adaptive update strategy which determined whether to implement template update network according to two quantitative indicators, so as to provide the optimal template for the next frame: one was the similarity between the tracking result of the current frame and the dynamic template; the other was the no reference evaluation result of the current frame image. The dual-template fusion module takes into account the advantages of the initial template and the dynamic template through the weighted fusion of the static template and dynamic template, which reduces the robustness of the tracker.

### 2.1. The feature extraction module and RPN

Two neural networks that shared weights formed a Siamese network for object feature extraction: One was the template branch for extracting features from the target template frame *TF* and the other was the search branch for extracting the characteristics of the search frame *SF*. The two branches shared parameters in a convolutional neural network.

The feature extraction module applied the AlexNet network used by the DaSiamRPN algorithm, and the RPN was also consistent with that of the DaSiamRPN algorithm. The feature map obtained by the feature extraction module was the input of RPN. RPN was used to obtain a more accurate target candidate box, including the classification branch φ_cls_(·) and the regression branch φ_*reg*_(·).

Firstly, the first frame image of the video was taken as the target template (as shown in the blue bounding box in the video frame in the upper left corner of Figure 1), then the anchor box was generated by the RPN, and the Softmax classifier was used to extract the positive anchors to obtain more accurate $A_{\text{W}\times H\times 2K}^{cls}$ in the classification branch. The classification branch determined whether each location was foreground or background:


(1) $$\begin{array}{l} A_{\text{W}\times H\times 2K}^{cls}\varphi_{\text{cls}}\left(SF\right)*\varphi_{cls}\left(TF\right) \end{array}$$

In Equation 1, $A_{\text{W}\times H\times 2K}^{cls}$ represents the classification feature map where the target and background score information of each predefined anchor box is stored. W and H represent the width and height of the feature map, respectively; K is the number of anchor boxes; *SF* represents the search frame; *TF* represents the template frame; and * refers to the cross-correlation operation.

Secondly, the non-maximum suppression (NMS) is used to determine the predefined anchor box (as shown in the green bounding box in the video frame at the lower right corner of Figure 1) from the feature map $A_{\text{W}\times H\times 2K}^{cls}$ obtained in the classification branch. x^*an*^, y^*an*^, w^*an*^, and h^*an*^ represent the center coordinates, width, and height of the predefined anchor box, respectively.

Then, the coordinate offset of the center point of the corresponding anchor box (*dx*^*reg*^,*dy*^*reg*^) and the length and width ratio of this anchor box to the real target box (*dw*^*reg*^, *dh*^*reg*^) are selected from the feature map obtained in the regression branch. The regression branch calculates all the target bounding boxes that may exist at each location:


(2) $$\begin{array}{l} A_{\text{W}\times H\times 4K}^{reg}\varphi_{\text{reg}}\left(SF\right)*\varphi_{reg}\left(TF\right) \end{array}$$

In Equation 2, $A_{\text{W}\times H\times 4K}^{reg}$ represents the regression feature map which stores the information such as the coordinate offset of the center point of the predefined anchor box and the width and height ratio of the predefined anchor box to the real target box.

Finally, the prediction box (as shown in the red bounding box in the video frame in the upper right corner of Figure 1) is obtained through the bounding box coordinate regression of the predefined anchor box. x^*pre*^ represents the abscissa of the center coordinate of the prediction box:


(3) $$\begin{array}{l} x^{pre}x^{an}+dx^{reg}\times w^{an} \end{array}$$

y^*pre*^ represents the ordinate of the center coordinate of the prediction box:


(4) $$\begin{array}{l} y^{pre}y^{an}+dy^{reg}\times h^{an} \end{array}$$

w^*pre*^ represents the width of the prediction box:


(5) $$\begin{array}{l} w^{pre}w^{an}\times e^{dw^{reg}} \end{array}$$

h^*pre*^ represents the height of the prediction box:


(6) $$\begin{array}{l} h^{pre}h^{an}\times e^{dh^{reg}} \end{array}$$

### 2.2. The dynamic template update module

#### 2.2.1. The UpdateNet network

**Figure 2:**
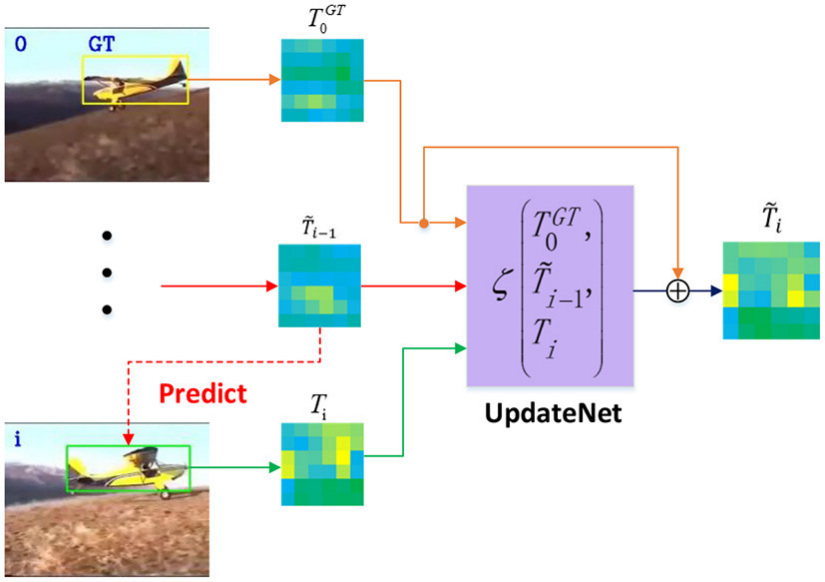
The network framework of UpdateNet.

The convolutional neural network of UpdateNet was used to implement the dynamic template update, and its network framework is shown in Figure 2 and can be represented by the following formula:


(7) $$\begin{array}{l} {\tilde{T}}_{i}=\zeta \left(T_{0}^{GT},{\tilde{T}}_{i-1},T_{i}\right) \end{array}$$

where ${\tilde{T}}_{i}$ is the cumulative template updated after the current frame tracking finished; ζ(·) is the function of the UpdateNet network; $T_{0}^{GT}$ is the template given in the first frame; ${\tilde{T}}_{i-1}$ is the dynamic template updated after the previous frame image target tracking finished; and *T*_*i*_ is the object tracking result for the current frame. A residual learning strategy is adopted by this network, which adds a jump connection from the template given in the first frame to the output. For the first frame of the object tracking image sequence, both *T*_*i*_ and ${\tilde{T}}_{i-1}$ are set to $T_{0}^{GT}$. New tracking results are used by UpdateNet to update the template to better adapt to the changes in the object appearance.

#### 2.2.2. Adaptive update strategy

The adaptive update strategy proposed in this paper determined whether to update dynamic templates by judging the necessity of updating the template and the possibility of template pollution if the template was updated according to two quantitative indicators: one was the similarity between each image region and the other was the no reference evaluation result of the image quality. In this way, it was able to suppress the adverse effects of noise interference and pollution of the template while adapting to the changes in the object appearance. The similarity between two image blocks was quantified by the *L*1 norm between them:


(8) $$\begin{array}{l} S={\left||\text{M}-\text{N}|\right|}_{1} \end{array}$$

In Equation 8, ||·|| is the *L*1 norm; M and *N* are the two image blocks whose similarity is compared; and *S* is the quantitative results of the similarity between the two image blocks. The smaller the value of *S*, the more similar the two image blocks are.

In this paper, NIQE (Natural Image Quality Evaluator; Mittal et al., 2013) was used to evaluate the image quality. The Mahalanobis distance between the MVG (Multivariate Gaussian Model) obtained through natural image feature fitting and that obtained through the feature fitting of the image to be measured is used by this method to indicate the image quality:


(9) $$\begin{array}{l} D=\sqrt{{\left({\text{v}}_{1}-v_{2}\right)}^{T}{\left(\frac{\sum_{1}+\sum_{2}}{2}\right)}^{-1}\left({\text{v}}_{1}-v_{2}\right)} \end{array}$$

where *D* represents the quantitative evaluation result of the image quality, and the smaller its value is, the better the image quality is. *v*_1_ and *v*_2_ represent the mean vectors of the MVG model of the natural image and that of the image to be tested, respectively. ∑_1_ and ∑_2_ are the covariance matrices of the MVG model of the natural image and that of the image to be tested, respectively.

**Figure 3:**
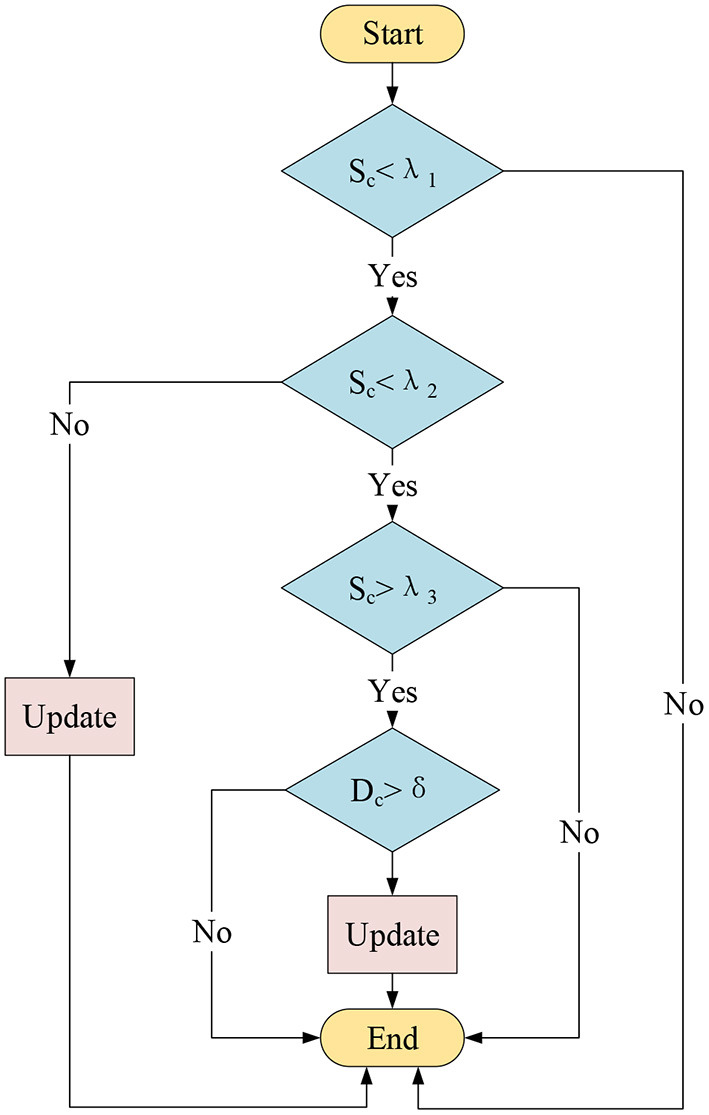
The adaptive update strategy.

With the above two quantitative evaluation indicators, the whole process of the proposed adaptive update strategy is shown in Figure 3. In order to accurately determine whether the template should be updated, a total of three similarity thresholds, λ_1_, λ_2_, λ_3_, and an image quality threshold, δ, are set. Among them, λ_1_ is the abnormal threshold value of the target appearance, and when the similarity between the tracking object of the current frame and the dynamic template *S*_*c*_ is > λ_1_, it indicates that the target appearance characteristics have changed beyond the normal range. λ_2_ is the obvious-change threshold value of the target appearance, and when *S*_*c*_ is > λ_2_, it indicates that the target appearance characteristics have changed significantly. λ_3_ is the changing threshold value of the target appearance and when *S*_*c*_ is > λ_3_, it indicates that there are some changes in the target appearance characteristics that have a certain impact on the target tracking, and conversely, the target appearance characteristics are almost unchanged. δ is the good image quality threshold, and when the quality evaluation result of the current target sub-image block *D*_*c*_ is < δ, it indicates that the target sub-image block of the current frame is high in quality and that the noise interference almost does not adversely affect the image acquisition.

In this process, the threshold value λ_1_ is first used to judge whether there is a serious abnormity in the current image acquisition and target tracking. If *S*_*c*_ is ≥ λ_1_, the dynamic template will not be updated to avoid the template being contaminated by abnormal data.

If *S*_*c*_ is < λ_1_, it is supposed to further judge whether it is ≥ λ_2_. If so, it indicates that the object appearance has been changed obviously under normal circumstances and in order to cope with these changes, the template must be updated.

If *S*_*c*_ is < λ_2_, it needs to continue to judge whether *S*_*c*_ is ≥ λ_3_. At this time, if *S*_*c*_ is smaller than λ_3_, it indicates that the object appearance feature is almost unchanged so there is no need to update the dynamic template. If *S*_*c*_ is ≥ λ_3_, it indicates that there are some changes in the object appearance under normal circumstances that have a certain impact on the target tracking, thought the changes are not significant enough to update the template. Then, there is a need to judge whether *D*_*c*_ is ≥ δ. If so, it indicates that the image is poor in quality so the dynamic template will not be updated to avoid template pollution, and conversely, the dynamic template will be updated.

### 2.3. “Dynamic-static” dual-template fusion module

The method in this paper fuses the feature map obtained by using static and dynamic templates in the RPN classification branch.


(10) $$A=\alpha A_{W\times H\times 2K}{}^{cls}+\left(1-\alpha \right){A^{\prime }}_{W\times H\times 2K}{}^{cls}$$

In Equation 10, *A* is the feature map of the classification branch after fusion and ${A^{\prime }}_{W\times H\times 2K}{}^{cls}$ represents the feature map obtained from the static template in the classification branch:


(11) $$\begin{array}{l} A_{\text{W}\times H\times 2K}^{\prime cls}\varphi_{\text{cls}}\left(SF\right)*\varphi_{cls}\left(TF_{0}\right) \end{array}$$

where $\varphi_{cls}\left(TF_{0}\right)$ represents the feature of the initial frame in the classification branch, and W, H, K, and *SF* have the same meaning as in Equation 11.

αϵ[0, 1], which represents the weight coefficient during fusion:


(12) $$\begin{array}{l} \alpha =\frac{S_{o}}{S_{d}+S_{o}} \end{array}$$

where *S*_*d*_ represents the quantization result of the similarity between the dynamic template of the previous frame and the object obtained by the previous frame tracking, and *S*_*o*_ is the similarity between the object obtained by the previous frame tracking and the templates of the initial frame.

The introduction of this module can give the object tracking algorithm the advantage of using static and dynamic templates, and make it suppress the adverse effects of noise interference and contamination of the template while adapting to the changes in the object appearance.

## 3. Experiment results and analysis

### 3.1. Implementation details and parameters

All the algorithms in this experiment were carried out using the Pytorch 1.7.1 deep learning platform on the deep learning workstation. The deep learning workstation uses the Windows 10 operating system with Intel^®^ Xeon(R) Gold 6139M CPU, 128 GB RAM, and the Nvidia GTX3080 GPU, which is equipped to perform parallel computing.

The ImageNet VID (**?**), YouTube—BoundingBoxes (Real et al., 2017), ImageNet DET, and COCO (Lin et al., 2014) datasets were used as training sets to train the feature extraction network to learn the similarity between objects. Ten sequences were randomly selected from the LaSOT (Fan et al., 2019) dataset as training sets to train the template update network. A batch of 64 samples were trained in each training phase, with a total of 50 epochs, and the learning rate of each epoch decreases logarithmically from 10^−7^ to 10^−8^. The stochastic gradient descent method with momentum was used for optimization, and in the actual optimization process, the difficulty of optimization gradually increased as the depth of feature extraction increased. In order to prevent the loss function of each layer from gradient explosion, the momentum was set to 0.9; the weight decay parameter was set to 0.0005; and the size of the template image and the search image were set to 127 × 127 and 271 × 271, respectively.

In addition, the average similarity between the tracking object of the current frame and the dynamic template *S*_*c*_ obtained by the training template update network was 0.0017, the variance was 0.0023, the standard deviation was 0.048, and the maximum value was 7. After many studies, we found that the threshold value of appearance abnormality λ_1_ should be more than four times the standard deviation. The threshold of appearance variation λ_2_ can be about 0.01 times the standard deviation. The appearance change threshold λ_3_ can be about 0.005 times the standard deviation. By calculating the image quality of more than 20,000 frames, we found that the image quality below 15 is significantly better. Therefore, when using the method in this paper, the thresholds λ_1_, λ_2_, λ_3_, and δ were set to 0.5, 6 × 10^−4^, 1 × 10^−4^, and 15.

### 3.2. Evaluation on benchmarks

#### 3.2.1. VOT2016 benchmark experiment

The VOT series dataset is one of the most used datasets for visual object tracking. The VOT2016 dataset includes 60 video sequences with multiple challenges, each of which is labeled with the visual properties of that sequence. There are six properties: camera motion, variation of light condition, occlusion, size change, motion change, and no degradation. The performance evaluation indicators used by the dataset include Expected Average Overlap (EAO), accuracy (A, Accuracy), and robustness (R, Robustness). Among them, EAO can better reflect the comprehensive performance of the tracking algorithm and is generally considered as the most important indicator. The accuracy, which describes the average overlap score, is obtained by calculating the ratio of intersection over union (IOU) of the prediction box and the truth box. Robustness is an indicator used to evaluate the stability of the tracking algorithm and the smaller its value is, the more stable the algorithm is.

**Figure 4:**
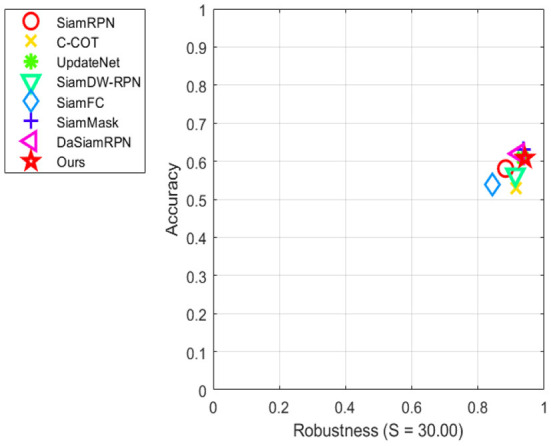
Accuracy-robustness ranking plots on the VOT2016 benchmark.

**Figure 5:**
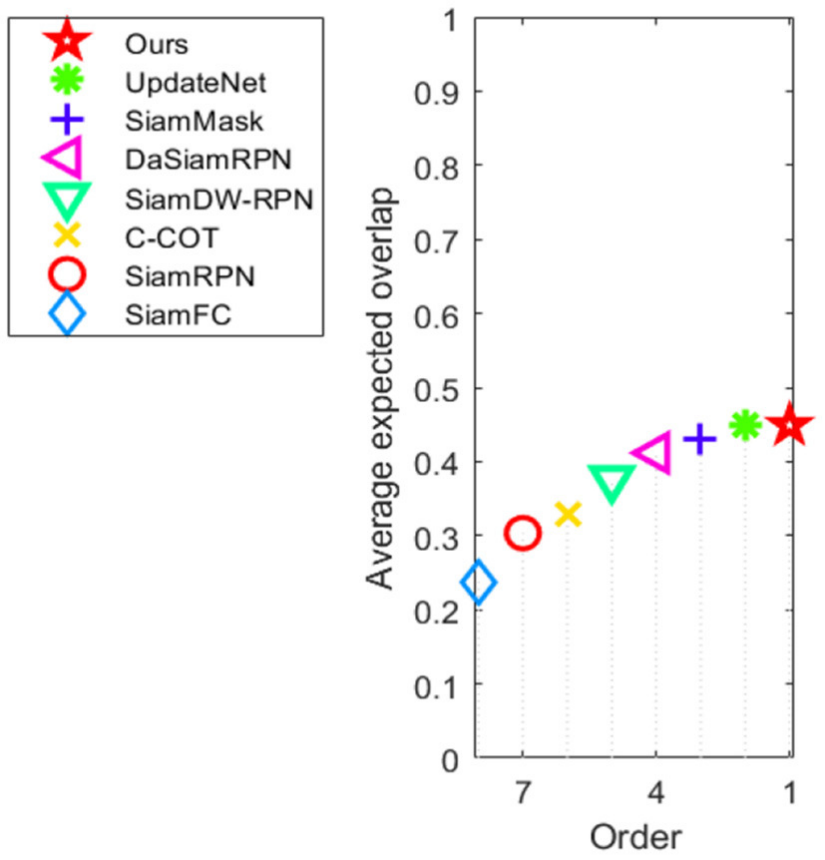
Expected overlap curves on the VOT2016 benchmark.

The performance of this method and the current mainstream object tracking algorithms (SiamFC, SiamRPN, SiamDW-RPN, SiamMask, C-COT, DaSiamRPN, and UpdateNet) in the VOT2016 dataset is shown in Table 1. As can be seen from the table, the robustness of this method has been greatly improved compared with other algorithms, at 0.183. This is 23% higher than the basic algorithm DaSiamRPN and 8.9% higher than the second-ranked UpdateNet algorithm. Its EAO score, which reflects the comprehensive performance of the object tracking algorithm, is also the highest, with a score of 0.449. It is 9.0% higher than the DaSiamRPN algorithm and 0.2% higher than the second-ranked UpdateNet algorithm. The accuracy rate ranks third among all algorithms, which is 1.8% lower than the DaSiamRPN algorithm and 3.4% lower than the SiamMask algorithm with the highest accuracy. Above all, the method in this paper has the best comprehensive performance among all the compared algorithms, especially in terms of robustness in complex environments. Figure 4 shows the visual sorting plot of accuracy and robustness, and Figure 5 shows the score sorting diagram of average overlap expectations.

**Table 1:** Performance comparison on the VOT2016 benchmark.

**Algorithms**	**EAO↑**	**A↑**	**R↓**
DaSiamRPN	0.412	0.620	0.238
UpdateNet	0.448	0.609	0.201
SiamFC	0.238	0.539	0.522
SiamRPN	0.303	0.581	0.387
SiamDW-RPN	0.376	0.565	0.281
SiamMask	0.430	0.630	0.206
C-COT	0.329	0.529	0.276
Ours	0.449	0.609	0.183

#### 3.2.2. OTB100 benchmark experiment

**Figure 6:**
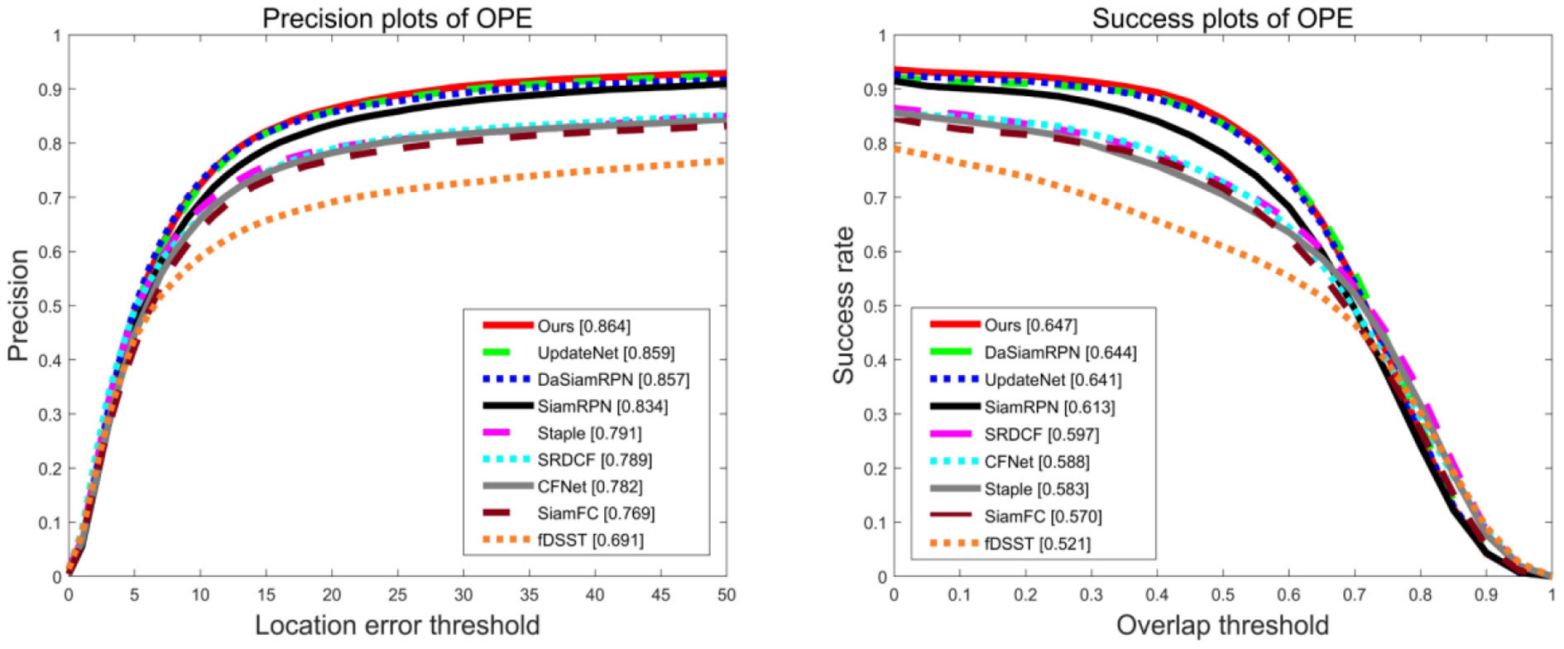
Precision and success plots of all compared trackers on OTB100.

The OTB100 dataset contains 100 video sequences, and the tracking scenes involved in these video sequences can be divided into 11 labeled properties. The two indicators, precision and success of tracking, are used by the dataset to evaluate the performance of the algorithm. Tracking precision refers to the ratio of the estimated number of frames with center position error to the total number of frames within 20 pixels, and the success refers to the percentage of the number of frames where the intersection over union of the target prediction box and the real bounding box is >0.5 to the total number of frames. The proposed method was compared with the current mainstream object tracking algorithms [SiamFC, SiamRPN, Staple (Bertinetto et al., 2016a), SRDCF (Danelljan et al., 2016b), CFNet (Valmadre et al., 2017), fDSST (Danelljan et al., 2017b), DaSiamRPN, and UpdateNet] on the OTB100 dataset, and Figure 6 shows the precision and success of each tracking algorithm. Among all the compared tracking algorithms, the proposed method ranks first, with a precision score of 86.4% and a success score of 64.7%. Compared with the basic algorithm DaSiamRPN, the precision and success of the proposed method increased by 0.8 and 0.4%, respectively. Compared with the UpdateNet algorithm, the precision and success of the proposed method increased by 0.6 and 0.9%, respectively. Overall, the performance of the proposed method was better than that of the current mainstream object tracking algorithms.

**Figure 7:**
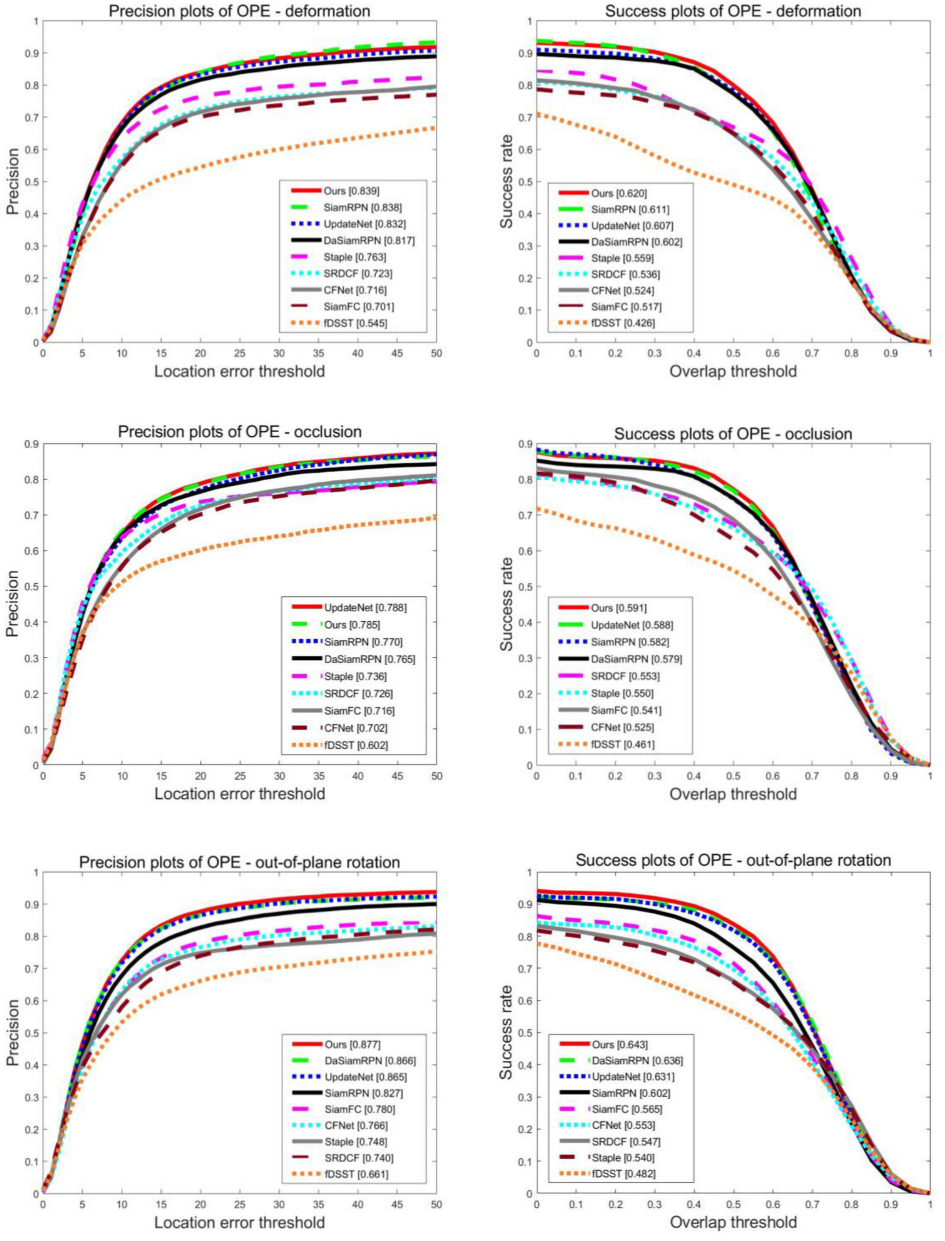
Precision and success plots of all compared trackers on deformation, occlusion, and out of plane rotation attributes on OTB100.

In particular, we present the comparison curves for deformation, occlusion, and out of plane rotation attributes on the OTB100 dataset in Figure 7. Obviously, in the precision plots, our tracking method outperformed all compared competitors on the deformation and out of plane rotation attributes. Compared with the UpdateNet algorithm, the precision increased by 0.8 and 1.4% and compared with the DaSiamRPN algorithm, the precision increased by 2.7 and 1.3%, respectively. However, in the occlusion attribute of the precision plots, our tracking method was 0.4% lower than the UpdateNet algorithm, but 2.6% higher than the DaSiamRPN algorithm. In the success plots, our tracking method obtained the highest success score on the deformation, occlusion, and out of plane rotation attributes. Compared with the UpdateNet algorithm, the precision increased by 2.1, 0.5, and 2.1%, and compared with the DaSiamRPN algorithm, the precision increased by 3.0 1.9, and 1.1%, respectively. It can be seen that our method can overcome challenges in object tracking.

### 3.3. Qualitative evaluation

**Figure 8:**
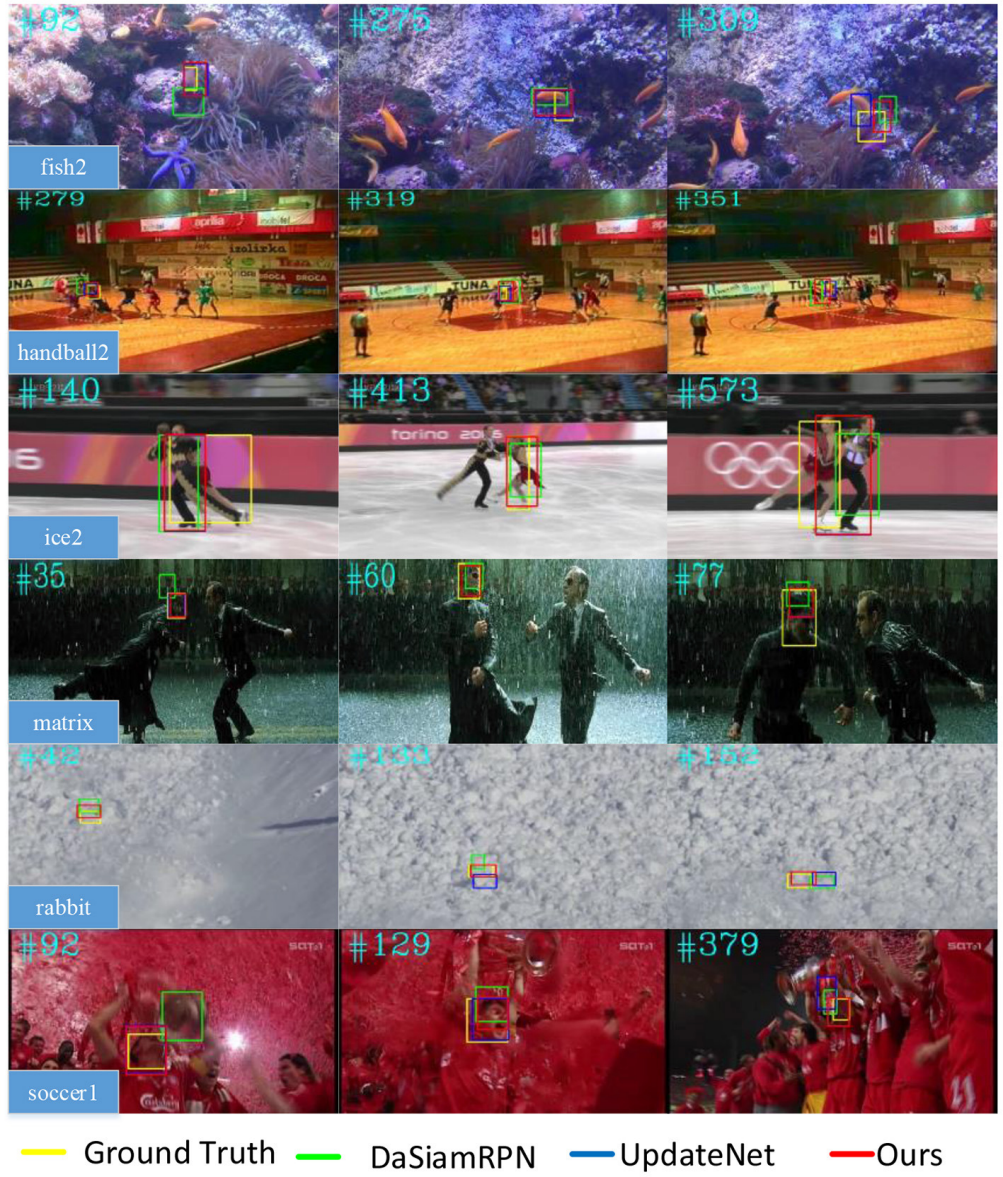
Qualitative evaluation on six challenging video sequences.

In order to visually display the tracking effect, several video sequences with various tracking difficulties were selected from the VOT2016 dataset and compared with the DaSiamRPN and UpdateNet algorithms to verify the experiment, as shown in Figure 8.

The main difficulty of tracking fish2 video sequences lies in the similar object interference. In the 92nd frame, the rapid movement of the object caused blurring, so the DaSiamRPN algorithm failed to track, while the method in this paper and the UpdateNet algorithm could track the object more accurately. In the 275th frame, there is an interfering object which is similar to the target, and the DaSiamRPN algorithm misjudged the analog as the tracking target. However, under the same conditions, the algorithm in this paper overcame the interference and achieved robust tracking. In the 309th frame, an interfering object which is similar to the target appears again, and the proposed method could track the target more accurately than the DaSiamRPN and UpdateNet algorithms.

The main difficulties of tracking handball2 video sequences are target occlusion, similar object interference, and posture changes. In the 279th frame, the tracking object is blocked by another moving object and due to the blurring of the target caused by its rapid movement, the DaSiamRPN algorithm tracked the target mistakenly. However, the proposed method could still track the object despite the occlusion caused by another moving object.

The main difficulties of tracking matrix video sequences are similar object interference and changes in light conditions. In the 35th frame, similar object interference appears, so the DaSiamRPN algorithm tracked the target mistakenly. In the 77th frame, the DaSiamRPN algorithm had a tracking drift due to the complex background and the significant changes in light conditions. However, the proposed algorithm could track the object more steadily in spite of the above difficulties.

The main difficulties of tracking the rabbit video sequences are the small size of the tracking object and similar background interference. In the 133rd and 152nd frames, both the DaSiamRPN algorithm and the UpdateNet algorithm failed to track the object, while the proposed method in this paper still located the object stably.

Under tracking object occlusion, similar target interference, and a complicated environment, the DaSiamRPN algorithm always uses the predefined target in the first frame as the template of the tracking in the subsequent frames. The feature information easily gets lost because of the changes in the object appearance, resulting in the inability to accurately obtain the position of the object. The UpdateNet algorithm uses the template update mechanism to update the templates of each frame, which can introduce noise polluting templates that then results in tracking failure. On the other hand, the “dynamic–static” dual-template fusion and dynamic template adaptive update method proposed in this paper has the advantages of using static and dynamic templates, and it can still present a good tracking performance despite target occlusion, similar object interference, image blurring, and changes in light conditions, etc.

### 3.4. Real-timeliness of tracking

The tracking speed of the proposed method and the DaSiamRPN and UpdateNet algorithms on the VOT2016 and OTB100 datasets are shown in Table 2. The DaSiamRPN algorithm can run at 110 frames per second (FPS) and 176 FPS on the VOT2016 and OTB100 datasets, respectively. The UpdateNet algorithm can only run at 76 and 79 FPS. Because the UpdateNet algorithm introduces the template update mechanism to improve performance, the tracking speed is significantly lower than DaSiamRPN algorithms, but it can still meet the requirements of real-time tracking. Our method achieved a running speed of 87 and 81 FPS on VOT2016 and OTB100 datasets, respectively, and the tracking speed was slightly higher than that of the UpdateNet algorithm. Unlike the UpdateNet algorithm, the adaptive update strategy proposed in this paper does not update the template dynamically in every frame, so it has better real-time performance.

**Table 2:** FPS comparison on the VOT2016 and OTB100 benchmark.

**Algorithms datesets**	**DaSiamRPN**	**UpdateNet**	**Ours**
VOT2016	110	76	87
OTB100	176	79	81

## 4. Conclusion

Deep learning object tracking algorithms based on Siamese networks have received extensive attention for their good performance in the testing of various benchmark tracking datasets. However, the earliest Siamese network object tracking algorithm uses the object position in the first frame image as a fixed template throughout the tracking, which cannot cope with the adverse effects of a change in the object appearance. The introduction of the template update mechanism makes the subsequent Siamese network better adapted to any change in the object appearance, though it also brings new problems, namely, causing the tracking template to be contaminated by external noise interference.

In order to further improve the performance of the target tracking algorithm, a Siamese tracker with “dynamic–static” dual-template fusion and dynamic template adaptive update is proposed in this paper, based on the DaSiamRPN Siamese network's object tracking and the UpdateNet template update network. The new method combines a static template and a dynamic one that is updated in real time for target tracking. An adaptive update strategy is applied when updating the dynamic template, which helps determine whether to update dynamic templates by judging the necessity of updating the template and the possibility of template pollution if the template is updated according to two quantitative indicators: one is the similarity between the tracking result of the current frame and the dynamic template; the other is the no reference evaluation result of the current frame image. In this way, it can suppress the adverse effects of noise interference and pollution of the template while adapting to the change in the target appearance. The experimental results showed that the robustness and EAO of the proposed method were 23 and 9.0% higher than those of the basic algorithm on the VOT2016 dataset, respectively, and that the precision and success were increased by 0.8 and 0.4% on the OTB100 dataset, respectively. The best comprehensive and real-time tracking performance was obtained on the above two large public datasets.

There are several important thresholds in the adaptive template update module of this method that make it necessary to collect samples in its application to statistical determination, which increases the difficulty of deploying the new method in the actual system. Therefore, the next step is to introduce an unsupervised machine learning algorithm so that self-adaption can be achieved.

## Data availability statement

The original contributions presented in the study are included in the article/Supplementary material, further inquiries can be directed to the corresponding author.

## Author contributions

DS: writing—original draft, data curation, and visualization. XW: supervision, conceptualization, and writing—review and editing. YM: methodology and software. ND and ZP: investigation and validation. All authors contributed to the article and approved the submitted version.
